# Modeling the effect of the number of presumptive colonies selected from agar plate on the recovery and prevalence of *E. coli* O157:H7 and non-O157 Shiga-toxin producing *E. coli* from dairy manure

**DOI:** 10.3389/fmicb.2026.1849872

**Published:** 2026-07-06

**Authors:** Xiaohong Wei, Megan Gaa, Nicole Tng, Tracee Da Silva, Tianchen Shan, Jessica E. Kim, Prachi Pandey, Xunde Li, Pramod K. Pandey, Edward R. Atwill

**Affiliations:** 1 Department of Population Health and Reproduction, University of California, Davis, Davis, CA, United States; 2 Western Center for Food Safety, University of California, Davis, Davis, CA, United States

**Keywords:** dairy manure, positive predictive value, presumptive colonies, prevalence, probability to be true positive, STEC

## Abstract

**Background:**

Although various laboratory protocols exist for the detection of Shiga toxin-producing *E. coli* (STEC), the number of presumptive colonies selected per sample for confirmation remains a critical yet largely overlooked factor that is has not been standardized between investigators.

**Objectives:**

Our study evaluated how the number of presumptive colonies selected for confirmation per fecal sample influences the probability to detect true positive samples, which when optimized can facilitate more accurate estimates of STEC prevalence in feces while simultaneously minimizing costs associated with confirming excessive numbers of colonies.

**Methods:**

An experimental trial was first conducted using inoculated concentrations of *E. coli* O157:H7 (−1.1 to 4.3 log₁₀ CFU/g) and non-O157 STEC (−1.9 to 4.6 log₁₀ CFU/g), with up to 45 presumptive colonies selected for qPCR confirmation. In parallel, a dairy fecal longitudinal survey was conducted on 14 dairy farms in California to examine how the number of presumptive colonies selected per sample affected STEC prevalence estimates, using the 45-presumptive colony protocol as the benchmark method. Using colony-based positive predictive values (c*PPV*) generated from the experimental trial for CT-SMAC, Rainbow Agar O157, and CHROMagar STEC selective agars, the hypergeometric (HG) and binomial (BN) distributions were used to calculate the probability of diagnosing a sample as true positive (Pr_HG_ and Pr_BN_, respectively) and the computation of the STEC prevalence for dairy fecal samples as a function of number of confirmed colonies.

**Results:**

The experimental trial comprised 126 spiked samples. Results showed that the high c*PPV* (~100%) values observed for all but the lowest bacterial concentrations (−1.1 log₁₀ CFU/g) for *E. coli* O157:H7 generated a > 90% probability of true positive detection (Pr_HG_ and Pr_BN_) when as few as 3 to 4 presumptive colonies were selected. Selecting only 3–4 presumptive colonies yielded a model-predicted prevalence of ~33%, representing a difference of <5% from the observed prevalence of 37.9%. This difference decreased to <2% when 8 (HG) or 11 (BN) presumptive colonies were selected. In contrast, for non-O157 STEC, ≥38 colonies per sample had to be selected when the maximum concentration of bacteria was ≤1.6 log₁₀ CFU/g in order to achieve ≥90% detection probability of true positives. Selecting 31 (HG) or 56 (BN) presumptive colonies resulted in a model-predicted prevalence of ~38%, which differed by 5% from the observed prevalence (42.9%). The difference was further reduced to <2% when 38 (HG) or 92 (BN) colonies were selected.

**Conclusion:**

In conclusion, given the large impact of colony selection totals on the Pr_BN_ or Pr_HG_ and STEC prevalence estimates, we recommend that authors report the number of presumptive colonies selected per sample in related future research in order to improve inter-study comparability and accurate interpretation of results from studies evaluating *E. coli* O157:H7 and especially non-O157 STEC in manure samples.

## 1 Introduction

*Escherichia coli* O157:H7 and non-O157 STEC are critical foodborne pathogens that pose a significant public health threat in the United States. These pathogens have been implicated in an average of 33 outbreaks of human illness every year over the past 5 years (2021–2025) (Center for Disease Control and Prevention, 2025). Infection with *E. coli* O157:H7 and non-O157 STEC can cause a range of clinical symptoms including diarrhea, abdominal pain, and nausea, with severe cases resulting in hemolytic uremic syndrome (HUS)-a condition related to kidney failure (Karmali et al., 1983; US Food and Drug Administration, 2019). Recent outbreaks illustrated the ongoing burden of *E. coli* O157:H7 and non-O157 STEC infections. For example, in October 2024, an outbreak of *E. coli* O157:H7 linked to slivered onions served on McDonald’s Quarter Pounder burgers resulted in 103 reported illnesses, including 34 hospitalizations and one death (US Food and Drug Administration, 2025a). Similarly, in December 2024, an outbreak of *E. coli* O121:H19 associated with organic carrots caused 48 reported cases, including 20 hospitalizations and one death, across multiple states (US Food and Drug Administration, 2025b). These outbreaks highlight the importance of enhanced food safety measures and accurate detection of *E. coli* O157:H7 and non-O157 STEC in diverse environmental matrices such as soil, sediment, wild and domestic animal feces during outbreak investigations and active surveillance.

The detection and recovery of *E. coli* O157:H7 and non-O157 STEC from different environmental matrices relies heavily on robust laboratory methods. Such methods typically involve initial enrichment in media, such as modified Buffered Peptone Water (mBPW), Tryptic Soy Broth (TSB), or MacConkey broth, to promote bacterial growth. This is followed by streaking the enriched samples onto selective media to isolate target organisms. Finally, presumptive colonies are confirmed using molecular techniques such as polymerase chain reaction (PCR) or other diagnostic tools to ensure high specificity (Arthur et al., 2004; Grant, 2005; Jarvis et al., 2010; European Committee for Standardization (CEN) et al., 2012; Cernicchiaro et al., 2013; Chui et al., 2013; Feng et al., 2018; Wei et al., 2023). To enhance the detection of STEC, protocols have been developed utilizing different enriching broths, selective media, and incubation conditions (e.g., varying times and temperatures) (Vimont et al., 2006). Also, various modifications have been introduced, such as adding supplements (e.g., acriflavine, washed sheep’s blood, Novobiocin) (Grant, 2005; Kase et al., 2015), traditional PCR, real-time PCR, immunomagnetic separation (Arthur et al., 2004; Antaki-Zukoski et al., 2021; Wei et al., 2023), and other advanced molecular methods (Li et al., 2020; Hu et al., 2021; Xia et al., 2022; Wang et al., 2024; Zhang et al., 2024).

Specifically, for isolating and detecting *E. coli* O157:H7 and non-O157 STEC, a variety of selective media agar are widely employed. For *E. coli* O157:H7 detection, commonly used media include CT-SMAC (MacConkey Agar with Sorbitol, Potassium tellurite, and Cefixime), Rainbow Agar O157, and CHROMagar O157 (Grant, 2005; Kilonzo et al., 2011; Feng et al., 2018). For non-O157 STEC, Rainbow Agar O157, CHROMagar STEC, MacConkey Agar, and Colorex O157 are frequently utilized (Zelyas et al., 2016; Feng et al., 2018). Despite these advancements, the critical factor of how many presumptive colonies should be selected per agar plate for confirmation has not been systematically standardized between investigators or scientific studies. For example, only a limited number of studies have reported the specific number of presumptive colonies chosen for confirmation per sample (e.g., up to 5, 6, or 10) while evaluating assay performance of selective media (Omisakin et al., 2003; Kase et al., 2015; Verhaegen et al., 2016; Jenkins et al., 2020; Lewis et al., 2020; Capps et al., 2021).

The binomial and hypergeometric distributions can both be used to model the probability of detecting a positive (a success) among a set of suspect samples. The binomial distribution assumes that the probability of success remains constant across independent trials, which implies sampling with replacement (Hogg et al., 2014). In the context of picking presumptive colonies from an agar plate, this assumption is generally unrealistic, as it would require repeatedly resampling the same colonies-a practice rarely performed in diagnostic microbiology. In contrast, the hypergeometric distribution is more suitable for this application because it accounts for sampling without replacement from a finite population of *N* presumptive colonies on the plate (Hogg et al., 2014). However, accurate estimation of *N* (total presumptive colonies) and *K* (true positive colonies) can be challenging when colony counts are too numerous to count or when selective media have low specificity. Fortunately, the probabilities derived from the binomial and hypergeometric distributions tend to converge when the sampling fraction is small (*n*/*N* < 0.1) and the population size is sufficiently large (*N* > 500).

Our study used the binomial and hypergeometric distributions in conjunction with spiked fecal samples of known bacterial concentration to characterize how the total number of presumptive colonies chosen for qPCR confirmation influences the probability of diagnosing a sample as true positive. The selective media used for this evaluation was CT-SMAC and Rainbow Agar O157 for *E. coli* O157:H7 detection and CHROMagar STEC for non-O157 STEC detection. Additionally, we conducted a longitudinal fecal survey on multiple dairy farms in order to characterize the impact of the number of presumptive colonies selected per sample on the estimated STEC prevalence, using the 45-presumptive colony protocol as a benchmark method. By addressing these critical knowledge gaps, this research seeks to optimize laboratory protocols for efficient bacterial pathogen detection and to provide evidence-based recommendations to improve the validity of making comparisons of pathogen prevalence between studies that use different numbers of presumptive colonies for confirmation from selective media.

## 2 Materials and methods

### 2.1 Experimental trial

#### 2.1.1 Preparation of inocula

**Figure 1:**
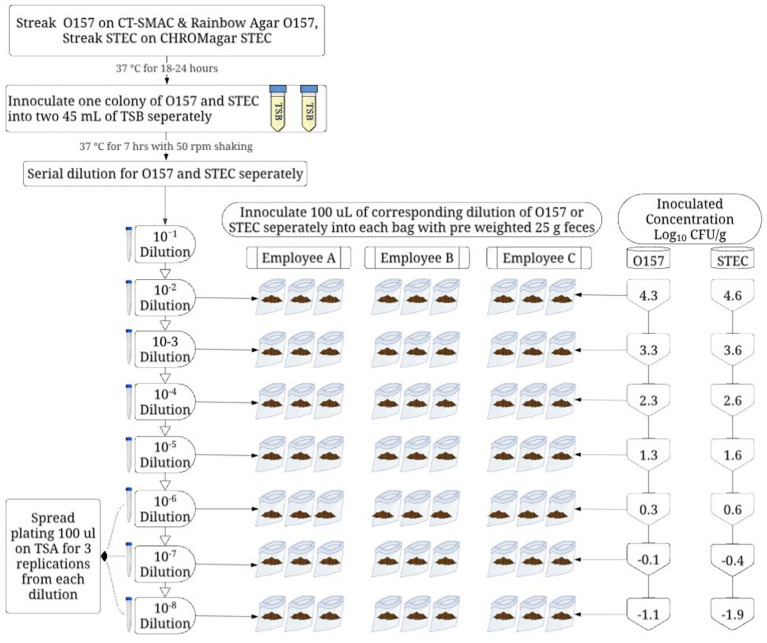
The process of inoculating *E. coli* O157:H7 and non-O157 STEC into dairy manure negative for these target pathogens. TSB, tryptic soy broth; CT-SMAC, MacConkey Agar with Sorbitol, Potassium tellurite, and Cefixime; O157 = *E. coli* O157:H7; STEC = non-O157 Shiga-toxin-producing *E. coli*; TSA, tryptic soy agar.

The inocula used in this study included two *E. coli* O157:H7 strains: one is American Type Culture Collection (ATCC) #43888 and the other one was isolated from a California dairy manure sample. In addition, we used three non-O157 STEC strains consisting of *E. coli* O74:H25 and *E. coli* O26:H11, both isolated from California dairy manure samples, and *E. coli* O103:H11 ATCC #BAA-2215. All strains were streaked onto their respective selective media: CT-SMAC (BD BBL, Sparks, MD, USA) and Rainbow Agar O157 (Biolog, Inc., Hayward, CA, USA) for *E. coli* O157:H7, and CHROMagar STEC (DRG International, Inc., Springfield, NJ, USA) for non-O157 STEC, both of which were incubated at 37 °C for 18–24 h. Pure colonies were selected from the corresponding media and inoculated into two separate 45 mL aliquots of TSB (Difco, BD, San Jose, CA, USA): one for *E. coli* O157:H7 and the other for non-O157 STEC. They were incubated in a shaking incubator at 25 °C for 2 h, followed by 42 °C for 8 h (Figure 1).

#### 2.1.2 Inoculation of *E. coli* O157:H7 and non-O157 STEC

The enriched TSB cultures were serially diluted in 9 mL phosphate-buffered saline (PBS; Sigma-Aldrich, St. Louis, MO, USA). To assess viable cell counts, three 100 μL aliquots of 10^−6^ to 10^−8^ dilutions were spread-plated in triplicate onto tryptic soy agar (TSA; Difco, BD, San Jose, CA, USA) and incubated at 37 °C for 18–24 h. One hundred μL of each dilution from 10^−2^ to 10^−8^ were correspondingly inoculated into each pre-weighed 25 g fecal samples (in Whirl-Pak bags) that were confirmed to be negative for both *E. coli* O157:H7 and non-O157 STEC. The seven inoculated concentrations for *E. coli* O157:H7 were −1.1, −0.1, 0.3, 1.3, 2.3, 3.3, and 4.3 log₁₀ CFU per gram, corresponding to 2, 22, 49, 493, 4,933, 49,333, 493,333 CFU per 25 grams, respectively. For non-O157 STEC, the inoculated concentrations were −1.9, −0.4, 0.6, 1.6, 2.6, 3.6, and 4.6 log₁₀ CFU per gram, corresponding to 0.3, 9, 97, 967, 9,667, 96,667, 966,667 CFU per 25 grams, respectively. A total of 126 inoculated samples were prepared as such: triplicate samples were processed by three different employees for seven concentrations for each set of bacterial pathogens (*E. coli* O157:H7 and non-O157 STEC): 3 replicated samples × 3 employees × 7 concentrations × 2 pathogen sets = 126 inoculated samples in total (Figure 1 displayed 63 inoculated samples for one pathogen as demonstration).

#### 2.1.3 Pathogen detection

**Figure 2:**
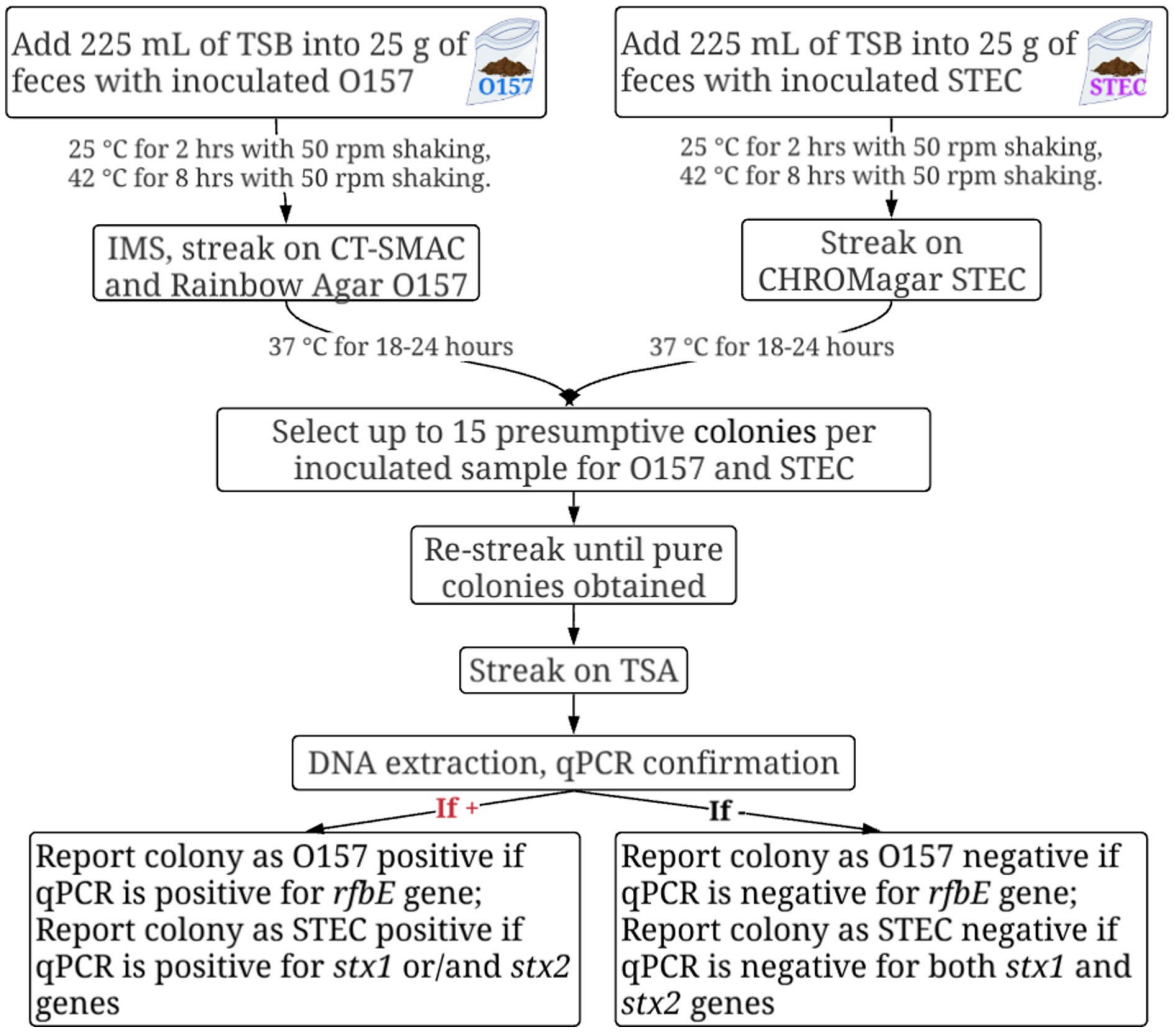
The flowchart of the laboratory methods for detecting *E. coli* O157:H7 and non-O157 STEC in dairy manure samples. TSB, tryptic soy broth; qPCR, quantitative PCR; CT-SMAC, MacConkey Agar with Sorbitol, Potassium tellurite, and Cefixime; O157 = *E. coli* O157:H7; STEC, non-O157 Shiga-toxin–producing *E. coli*; TSA, tryptic soy agar; IMS, immunomagnetic separation; − represents negative, and + represents positive.

The laboratory methods for examining *E. coli* O157:H7 and non-O157 STEC were based on our previously established protocols with slight modifications to accommodate the different matrices used in this study (Barkocy-Gallagher et al., 2002; Wei et al., 2023). Briefly, for all 126 inoculated samples, 225 mL of TSB was added to 25 g of pre-weighed manure inoculated with the corresponding concentrations of *E. coli* O157:H7 or non-O157 STEC (Figure 2). After incubation, a 10 μL loop of the enrichment was streaked onto CHROMagar STEC for non-O157 STEC. For *E. coli* O157:H7, 100 μL of bead suspension from immunomagnetic separation (IMS) was streaked onto CT-SMAC and Rainbow Agar O157 (50 μL per plate). For each triplicate inoculated sample at the specified concentration, up to 15 presumptive colonies (45 per employee and concentration) were re-streaked, purified, and confirmed through DNA extraction and quantitative PCR (qPCR) analysis to detect *rfbE* gene for *E. coli* O157:H7 and *stx*1/*stx*2 genes for non-O157 STEC (Atwill et al., 2015; Baker et al., 2019; Cooley et al., 2007). The primer and probe sequences, together with the full assay conditions, are provided in Table 1. The qPCR cutoff value was set at a cycle threshold (Ct) of 30. All qPCR data were analyzed using QuantStudio™ 3 Real-Time PCR System (Applied Biosystems™, Thermo Fisher Scientific, Waltham, MA, USA).

**Table 1:** The primer and probe sequences, together with the full assay conditions, for the detection of *stx* and *rfbE* genes.

Primer/probes (concentration: 100 μM) and master mix		Volume (μL)	Reaction parameters
*stx*1-F	5′- CATCGCGAGTTGCCAGAAT -3′	0.06	50 °C for 2 min, 95 °C for 20 s, 40 cycles of 95 °C for 3 s and 60 °C for 30 s
*stx*1-R	5′- TCCCACGGACTCTTCCATCT -3′	0.06	
*stx*1-P	5’FAM- ATCTGATGATTTCCTTCTATGTGTCCG -BHQ-1 3′	0.05	
*stx*2-F	5′- GGACCACATCGGTGTCTGTTATT -3′	0.06	
*stx*2-R	5′- CCCTCGTATATCCACAGCAAAAT -3′	0.06	
*stx*2-P	5’CAL Fluor Gold 540- CCACACCCCACCGGCAGT -BHQ-1 3′	0.05	
TaqMan™ Environmental Master Mix		10.00	
UltraPure™ DNase/RNase-free distilled water		7.66	
Sample DNA		2.00	
*rfbE*-F	5′- TTTCACACTTATTGGATGGTCTCA -3′	0.04	95 °C for 10 min, 40 cycles of 95 °C for 15 s and 60 °C for 1 min
*rfbE*-R	5′- TGAGTTTATCTGCAAGGTGATTCC -3′	0.04	
*rfbE*-P	5′ 6-FAM-TTCTAACTAGGACCGCAGAGGAAAGAGAGGAATTA-BHQ-1 3′	0.04	
TaqMan™ Environmental Master Mix		10.00	
UltraPure™ DNase/RNase-free distilled water		7.88	
Sample DNA		2.00	

F is forward; R is reverse; P is probe; min is minutes; s is seconds.

Presumptive colonies were selected according to the manufacturer’s instructions for each selective media. Colony color and morphology served as the primary criteria for identification. Suspected *E. coli* O157:H7 colonies appeared purple-blue on Rainbow Agar O157 and colorless on CT-SMAC. On CHROMagar STEC, typical non-O157 STEC colonies were mauve, with or without fluorescence (Hardy Diagnostics, 2020; Biolog, 2024; CHROMagar STEC, 2024). All laboratory personnel involved in colony selection received training prior to the present study. Presumptive colonies were selected based on colony color and morphology, following the manufacturer’s instructions for each selective media. To ensure consistency and minimize single-person bias, selections were performed by three trained employees under blinded conditions. Employees were unaware of the inoculation concentration; only the sample ID was known. Selections were cross-checked among the three employees, and any disagreements were resolved through discussion until consensus was reached.

The required sample size of colonies per sample for each unique combination of experimental conditions (e.g., pathogen group, bacterial concentration) was calculated using the following assumptions and methods: (1) expected proportion (*p*) = 0.13 of presumptive colonies that would confirm positive (e.g., PPV) for CHROMagar STEC (Zelyas et al., 2016); (2) *Z* = 1.96 (corresponding to a 95% confidence interval); (3) margin of error (*E*) = 0.10. Using these values in the standard formula for an infinite population, $n=\frac{Z^{2}p(1-p)}{E^{2}}$ (Hogg et al., 2014), yielded an approximate integer value of 43. However, considering the practical distribution of colonies in laboratory work and to ensure sufficient coverage, we conservatively increased this to 45 colonies per sample. The same sample size applied to the dairy fecal longitudinal survey.

### 2.2 Dairy fecal longitudinal survey

From December 2023 to June 2024, 140 composite dairy manure samples were collected from 14 dairy farms in central California. Each composite sample consisted of five individual samples in equal portions and homogenized thoroughly. The methods for detecting *E. coli* O157:H7 or non-O157 STEC were consistent with those described in the experimental trial (Figure 2), with minor adjustment: three employees together selected up to 45 presumptive colonies per sample for analysis (up to 15 colonies per employee) for both *E. coli* O157:H7 or non-O157 STEC. For each employee, 25 g of fecal material from each composite sample was enriched in 225 mL of TSB. The remaining steps followed the same procedures described in Figure 2, including plating on selective media, colony purification, confirmation through DNA extraction and qPCR analysis targeting *rfbE* gene for *E. coli* O157:H7 and *stx*1/*stx*2 genes for non-O157 STEC.

### 2.3 Statistical and probability analyses

#### 2.3.1 Colony-based PPV calculation

**Figure 3:**
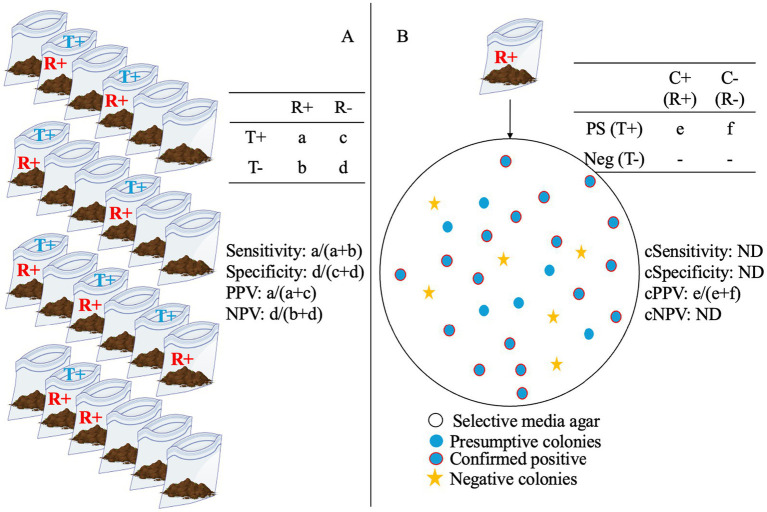
Illustration of the calculations of sensitivity, specificity, positive predictive value, and negative predictive value using both sample-based **(A)** and colony-based **(B)** approaches. R + is true positive; R − is true negative; T + is test positive; T − is test negative; PS is presumptive colonies; Neg is negative colonies; C + is qPCR confirmed positive; C − is qPCR confirmed negative; ND is not determined; cSensitivity is colony-based sensitivity; cSpecificity is colony-based specificity; cPPV is colony-based positive predict value; cNPV is colony-based negative predict value.

The definition of Sensitivity *Se*, Specificity *Sp*, *PPV*, and negative predictive value (*NPV*) are traditionally based on sample-level assessments (Gordis, 2013). The standard calculations are presented in Figure 3A. For instance, *PPV* is typically calculated as the number of true positive samples (a) divided by the total number of test positive samples (a + c) (Gordis, 2013). For our study, *PPV* was determined at the bacterial colony level (colony-based *PPV*) in order to model how the total number of presumptive colonies selected for qPCR confirmation influenced measures such as the *PPV* of an assay. Specifically, based on the color and morphology of colonies on specific selective media, colonies were classified as presumptive or negative. Being presumptive for a colony was equivalent to being test positive on selective media. Only presumptive colonies were selected for purification and qPCR confirmation; those colonies confirmed as positives were defined as true positives. As illustrated in Figure 3B, colony-based *PPV* was calculated as the number of qPCR-confirmed positive colonies (e) divided by the total number of presumptive colonies (e + f). Since negative colonies based on color and morphology were not subjected to qPCR confirmation, colony-based *Se*, *Sp*, and *NPV* were not determined and therefore not the focus of this study.

To evaluate assay performance of the combined use of Rainbow Agar O157 and CT-SMAC for identifying *E. coli* O157:H7 and CHROMagar STEC for detecting non-O157 STEC, the colony-based *PPV* was calculated for each bacterial pathogen. In our laboratory, we utilized three employees to process each fecal sample for bacterial detection given the workload of selecting and processing up to 45 presumptive colonies for each sample. As a consequence, the colony-based *PPV* was first determined for each inoculated concentration at the individual employee level (c*PPV*_employee_) using Equation 1 and then averaged across all three employees (c*PPV*_mean_) using Equation 2. For each individual sample from the longitudinal dairy fecal survey, colony-based *PPV* was calculated using Equation 3 (c*PPV*_sample_).


$$c\mathrm{PP}V_{\text{employee}}=\frac{\begin{array}{c} \mathrm{Sum}\;\text{of presumptive colonies comfirmed}\\ \text{positive}\;\mathrm{by}\;\text{qPCR}\;\mathrm{per}\;\text{employee} \end{array}}{\begin{array}{c} \mathrm{Sum}\;\text{of selected presumptive}\\ \text{colonies}\;\mathrm{per}\;\text{employee} \end{array}}$$ (1)

$$c\mathrm{PP}V_{\text{mean}}=\frac{\mathrm{Sum}\;\text{of}\;c\mathrm{PP}V_{\text{employee}}}{\text{Number of employees}}$$ (2)

$$c\mathrm{PP}V_{\text{sample}}=\frac{\begin{array}{c} \mathrm{Sum}\;\text{of presumptive colonies}\\ \text{comfirmed}\;\mathrm{by}\;\text{qPCR}\;\mathrm{per}\;\text{sample} \end{array}}{\begin{array}{c} \mathrm{Sum}\;\text{of selected presumptive}\\ \text{colonies}\;\mathrm{per}\;\text{sample} \end{array}}$$ (3)

#### 2.3.2 Calculation of the probability of diagnosing a sample as true positive when selecting different numbers of presumptive colonies

A fecal sample was defined as true positive if at least one selected presumptive colony was confirmed positive by qPCR. The probability of diagnosing a sample as true positive was then equivalent to the probability of observing at least one of the selected presumptive colonies as qPCR-confirmed positive (*x* > 0) and can be calculated using the cumulative distribution function (CDF) of either the binomial distribution (BN, Equation 4) or hypergeometric distribution (HG, Equation 5), depending on one’s assumptions and application. The binomial distribution requires three parameters in the CDF to calculate the probability (Pr_BN_) (Hogg et al., 2014): (1) probability (*p*): represented by c*PPV*_mean_ or c*PPV*_sample_, which is an estimate of the probability for a selected presumptive colonies to confirm positive by qPCR, derived as the total number of qPCR-confirmed positive colonies among all selected presumptive colonies; (2) number of trials (*n*): the number of presumptive colonies selected for confirmation; (3) number of successes (*x*) out of (*n*): the number of selected presumptive colonies confirmed as positive by qPCR. The hypergeometric distribution uses four parameters for its CDF to calculate the probability of being a true positive (Pr_HG_) (Hogg et al., 2014): (1) population size (*N*): in this study, *N* would be the total number of presumptive colonies per agar plate when not too numerous to count; (2) number of successes (*K*) in the population (*N*): the number of presumptive colonies *N* that would be confirmed positive by qPCR if all *N* colonies are subject to confirmation; (3) number of draws (*n*): the number of presumptive colonies selected (*n*) for confirmation per sample if a laboratory constrained by funding, time or labor such that *n* < *N*; (4) number of observed successes (*x*) out of (*n*): the number of selected presumptive colonies confirmed positive by qPCR.


$${\mathrm{Pr}}_{\mathrm{BN}}=f(0<x\leq n)=\sum_{x=1}^{n}(\begin{array}{c} n\\ x \end{array})p^{x}{\left(1-p\right)}^{n-x}$$ (4)

$${\mathrm{Pr}}_{\mathrm{HG}}=f(0<x\leq n)=\sum_{x=1}^{n}\frac{\left(\begin{array}{c} N\\ x \end{array})(\begin{array}{c} N-K\\ n-x \end{array}\right)}{\left(\begin{array}{c} N\\ n \end{array}\right)}$$ (5)

#### 2.3.3 Estimation of fecal prevalence of *E. coli* O157:H7 and non-O157 STEC as a function of the number of presumptive colonies (*x*) selected for confirmation per sample

Resources such as funding, time or labor are often constrained in research or scientific endeavors, leading to the selection of a just a subset of all presumptive colonies per agar plate for bacterial confirmation. For example, when only a few presumptive colonies (*x* = 1 to 4) are selected for confirmation per agar plate, the likelihood of missing a true positive colony becomes greater. This reduction in the Pr_BN_ or Pr_HG_ due to selecting fewer presumptive colonies is especially problematic when using selective media with low specificity, which can result in numerous false positive colonies and low values for *cPPV*_sample_. Alternatively, selecting large numbers of presumptive colonies for confirmation (i.e., *x* > 50) may be a waste of resources when the values of *cPPV* are reasonably large, leading to Pr_BN_ or Pr_HG_ approximating 100% with far fewer colonies. How to optimize this decision is in part the rationale for the following calculations regarding the minimal number of presumptive colonies to select for confirmation yet still achieve an accurate estimate of the prevalence of STEC in a set of fecal samples.

In order to test these assertions, we collected 140 fecal samples from 14 dairy farms as a case study to model the effect of choosing up to 45 or more presumptive colonies (*x*) for qPCR confirmation on the observed prevalence of *E. coli* O157:H7 and non-O157 STEC in these samples. From a practical perspective, 45 colonies represented a reasonably intense effort to identify qPCR-positive colonies per fecal sample and was suitable for the modeling efforts described immediately below. For each qPCR-confirmed positive fecal sample (i.e., at least one colony was qPCR-positive (0 < *x* ≤ 45) per fecal), we calculated the *cPPV*_sample(i)_ and used this value to then calculate Pr_BNi_ and Pr_HGi_ when *x*_i_ = 1, or *x*_i_ = 2, … up to *x*_i_ = 45 colonies depending on availability. All negative samples were assigned a value of 0 for Pr_BNi_ and Pr_HGi_ given the absence of presumptive colonies and/or no qPCR-positive colonies. Finally, for each scenario of *x* equal to 1 through 45, we summed the 45 values of Pr_BNi_ and Pr_HGi_ from each fecal sample to generate a model predicted fecal prevalence for this set of 140 fecal samples under the differing scenarios of *x*_i_ = 1, 2, … 45 (Equations 6, 7). Table 2 is an example of using Equations 6, 7 to calculate the fecal prevalence_BN_ and prevalence_HG_ using five mock fecal samples, four of which were positive and the fifth being negative.


$${\text{Fecal prevalence}}_{\mathrm{HG}}=\frac{\mathrm{Sum}\;\text{of each fecal sample}\prime s\;{\mathrm{Pr}}_{\mathrm{HGi}}}{\text{Total number of manure samples}}$$ (6)

$${\text{Fecal prevalence}}_{\mathrm{BN}}=\frac{\mathrm{Sum}\;\text{of each fecal sample}\prime s\;{\mathrm{Pr}}_{\mathrm{BNi}}}{\text{Total number of manure samples}}$$ (7)

**Table 2:** Example calculation^a^ of fecal prevalence of *E. coli* O157:H7 based on the probability of being a true positive, using binomial (BN) and hypergeometric (HG) methods, as applied to five fecal samples from the dairy cattle longitudinal survey.

No. of selected presumptive colonies	Sample ID_i_	Total number of presumptive colonies (M)	Number of qPCR positive colonies (R)	*cPPV_sample(i)_ (R/M)*	Pr_BNi_	Pr_HGi_	Fecal prevalence_BNi_ (sum of Pr_BNi_/5)	Fecal prevalence_HGi_ (sum of Pr_HGi_/5)
1	1	45	1	0.022	0.022	0.022	37.3%	37.4%
	2	45	12	0.267	0.267	0.267		
	3	19	11	0.579	0.579	0.579		
	4	3	3	1	0.999	1		
	5	0	0	0	0	0		
5	1	45	1	0.022	0.106	0.111	57.6%	58.2%
	2	45	12	0.267	0.788	0.806		
	3	19	11	0.579	0.987	0.995		
	4	3	3	1.000	0.999	1		
	5	0	0	0	0	0		
19	1	45	1	0.022	0.348	0.422	66.9%	68.4%
	2	45	12	0.267	0.997	0.999		
	3	19	11	0.579	0.999	1		
	4	3	3	1	0.999	1		
	5	0	0	0	0	0		
45	1	45	1	0.022	0.636	1	72.7%	80.0%
	2	45	12	0.267	0.999	1		
	3	19	11	0.579	0.999	1		
	4	3	3	1	0.999	1		
	5	0	0	0	0	0		

cPPV is colony-based positive predictive value. BN is binomial distribution; HG is hypergeometric distribution.

a Calculations based on a mock 80% observed prevalence (4/5 positives) derived from using the 45-presumptive colony protocol.

#### 2.3.4 Conditions for convergence between hypergeometric and binomial predictions for the probability of diagnosing a sample as true positive

Lastly, the final goal of this study was to determine under what conditions of *N* (total presumptive colonies per agar plate) and *n* (selected presumptive colonies) does convergence of Pr_BN_ and Pr_HG_ occur, thereby making the concern over choosing the binomial versus hypergeometric distribution due to differences in their assumptions immaterial if the resulting prevalence estimates are approximately equivalent. We modeled two different scenarios that we encounter when the targeted pathogen is either *E. coli* O157:H7 or non-O157 STEC. Scenario 1 (high *cPPV*): the selective media exhibits relatively high specificity with few false presumptive colonies. Scenario 2 (low *cPPV*): the selective media exhibits low specificity which can generate large numbers of presumptive colonies to choose from with many being false positives, typical of non-O157 STEC agar. For both scenarios, we identified the smallest *N* and *n* values that resulted in a maximum allowable difference between Pr_BN_ and Pr_HG_ to be ≤2% as an example of high convergence between both model’s predictions. If both probability models predict approximately the same value for a set of conditions, then we consider the value to be relatively robust to distributional assumptions.

## 3 Results

### 3.1 Recovery of the selected presumptive colonies across different inoculation concentrations

**Figure 4:**
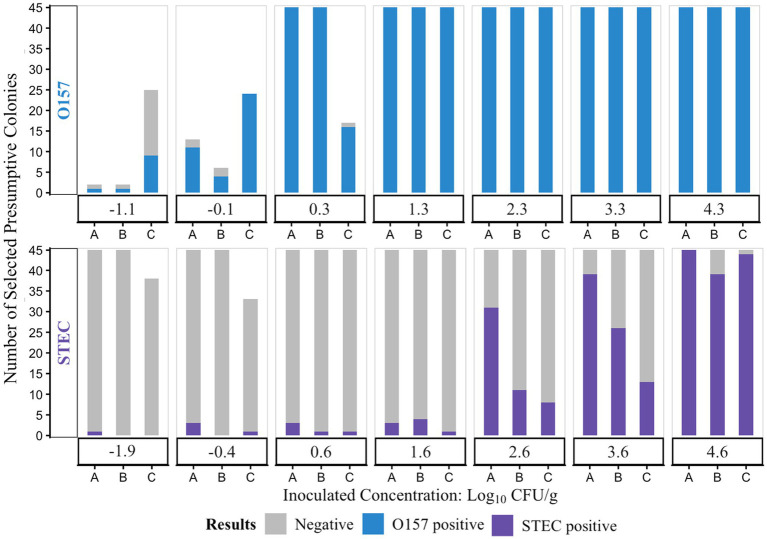
Results for presumptive colonies selected by each of three employees across different concentrations for *E. coli* O157:H7 from CT-SMAC and Rainbow Agar O157 combined, and for non-O157 STEC from CHROMagar STEC. O157 = *E. coli* O157:H7; STEC = non-O157 Shiga-toxin–producing *E. coli*; CT-SMAC = MacConkey Agar with Sorbitol, Potassium tellurite, and Cefixime; A, B and C are employee A, employee B and employee C, respectively.

For *E. coli* O157:H7, all three employees successfully recovered at least one positive colony from every concentration (Figure 4), even from the lowest concentration of −1.1 log₁₀ CFU/g (~2 CFU/25 g). In contrast, for non-O157 STEC, at −1.9 log₁₀ CFU/g (~0.3 CFU/25 g) and −0.4 log₁₀ CFU/g (~9 CFU/25 g), only two of the three employees recovered positive colonies, even after selecting up to 45 presumptive colonies (Figure 4). However, at inoculated concentrations equal to or higher than 0.6 log₁₀ CFU/g (≥97 CFU/25 g), all three employees successfully recovered at least one qPCR-confirmed positive colony. Additionally, for *E. coli* O157:H7, there were fewer presumptive colonies per selective media plate at lower concentrations (−1.1 and −0.1 log₁₀ CFU/g) compared to higher concentrations (>0.3 log₁₀ CFU/g), regardless of the employee, suggesting higher levels of specificity (i.e., few false presumptive colonies). Conversely, for non-O157 STEC, the available presumptive colonies per agar plate consistently exceeded 45 colonies, regardless of the employee or concentration, suggesting lower levels of specificity (i.e., multiple false presumptive colonies).

### 3.2 Colony-based *PPV* across different inoculation concentrations

**Figure 5:**
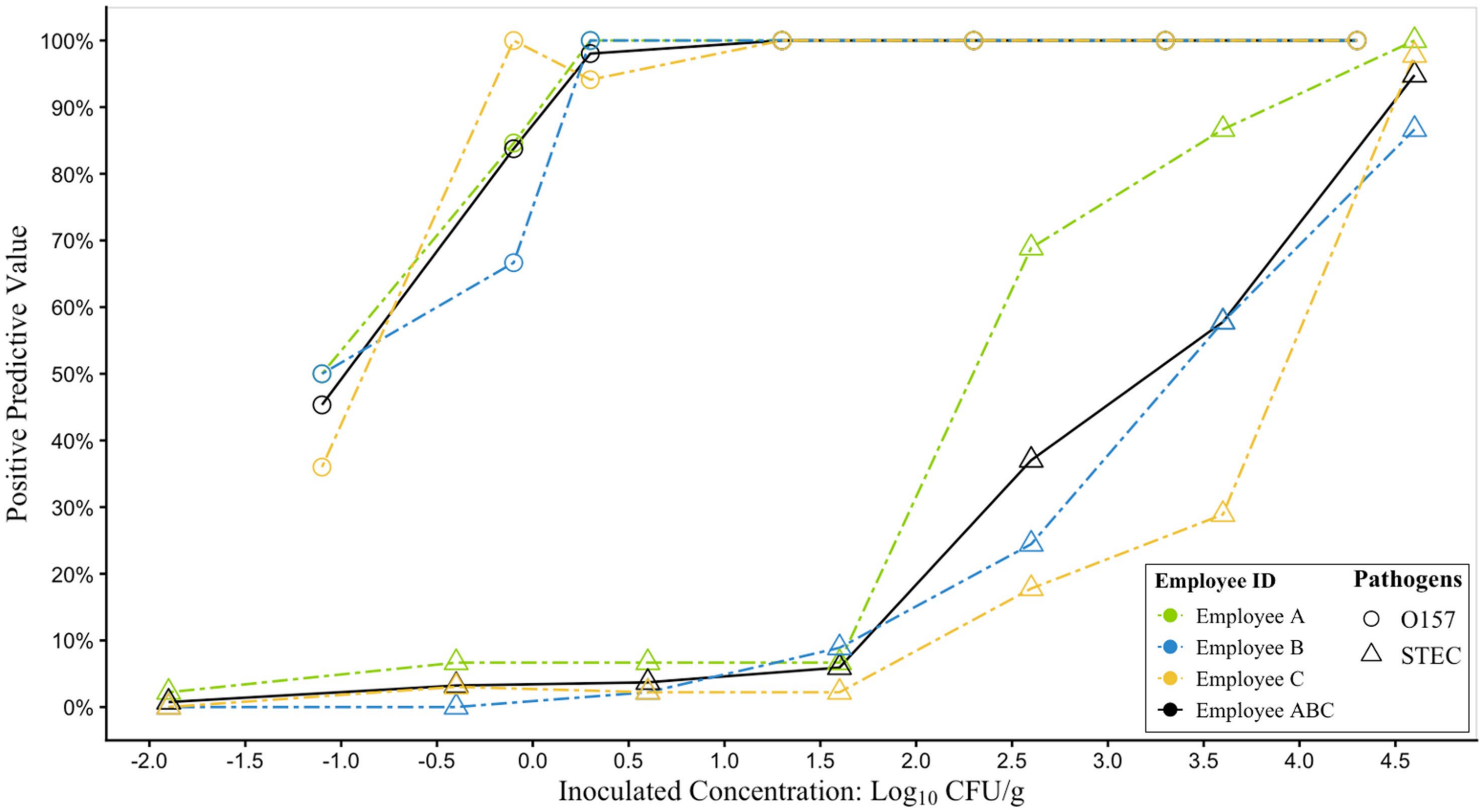
The colony-based positive predictive value for each of three individual employees and the arithmetic mean of the three employees based on selecting up to 45 presumptive colonies for confirmation for *E. coli* O157:H7 on CT-SMAC and Rainbow Agar O157 combined, and for STEC on CHROMagar STEC across varying concentrations. O157 = *E. coli* O157:H7; STEC = non-O157 Shiga-toxin–producing of *E. coli*; CT-SMAC = MacConkey Agar with Sorbitol, Potassium tellurite, and Cefixime; CI, confidence interval, shown as gray bars in the figure, are for the arithmetic mean.

The c*PPV*_employee_ and c*PPV*_mean_ at varying inoculated concentrations of *E. coli* O157:H7 and non-O157 STEC are shown in Figure 5. Regarding *E. coli* O157:H7, the dose–response curve for c*PPV*_employee_ and c*PPV*_mean_ was 35 to 50% at the lowest concentration of −1.1 log₁₀ CFU/g and quickly reached an asymptote of approximately 100% once the bacterial concentration reached ≥0.3 log₁₀ CFU/g. The 95% confidence intervals (CIs) were calculated for the c*PPV*_mean_, which reflect the degree of agreement or disagreement regarding *cPPV* values between the three employees. For example, the confidence intervals increased considerably at higher concentrations for non-O157 STEC, but were considerably more narrow for *E. coli* O157:H7. This pattern indirectly reflects the high specificity of the selective media used for *E. coli* O157:H7. In contrast, the c*PPV*_mean_ values for non-O157 STEC were under 10% for concentrations ≤1.6 log₁₀ CFU/g and only reached an asymptote of approximately 95% at the highest concentrations of 4.6 log₁₀ CFU/g. The 95% CIs were widest in the mid-range concentrations (2.6–3.6 log₁₀ CFU/g). These results indicate the relatively low specificity of CHROMagar STEC for non-O157 STEC, as well as the considerable uncertainty in the visual identification of presumptive colonies based on colony and morphology.

### 3.3 Modeling the effect of inoculation concentration and number of presumptive colonies selected for confirmation on the probability of diagnosing a sample as true positive

**Figure 6:**
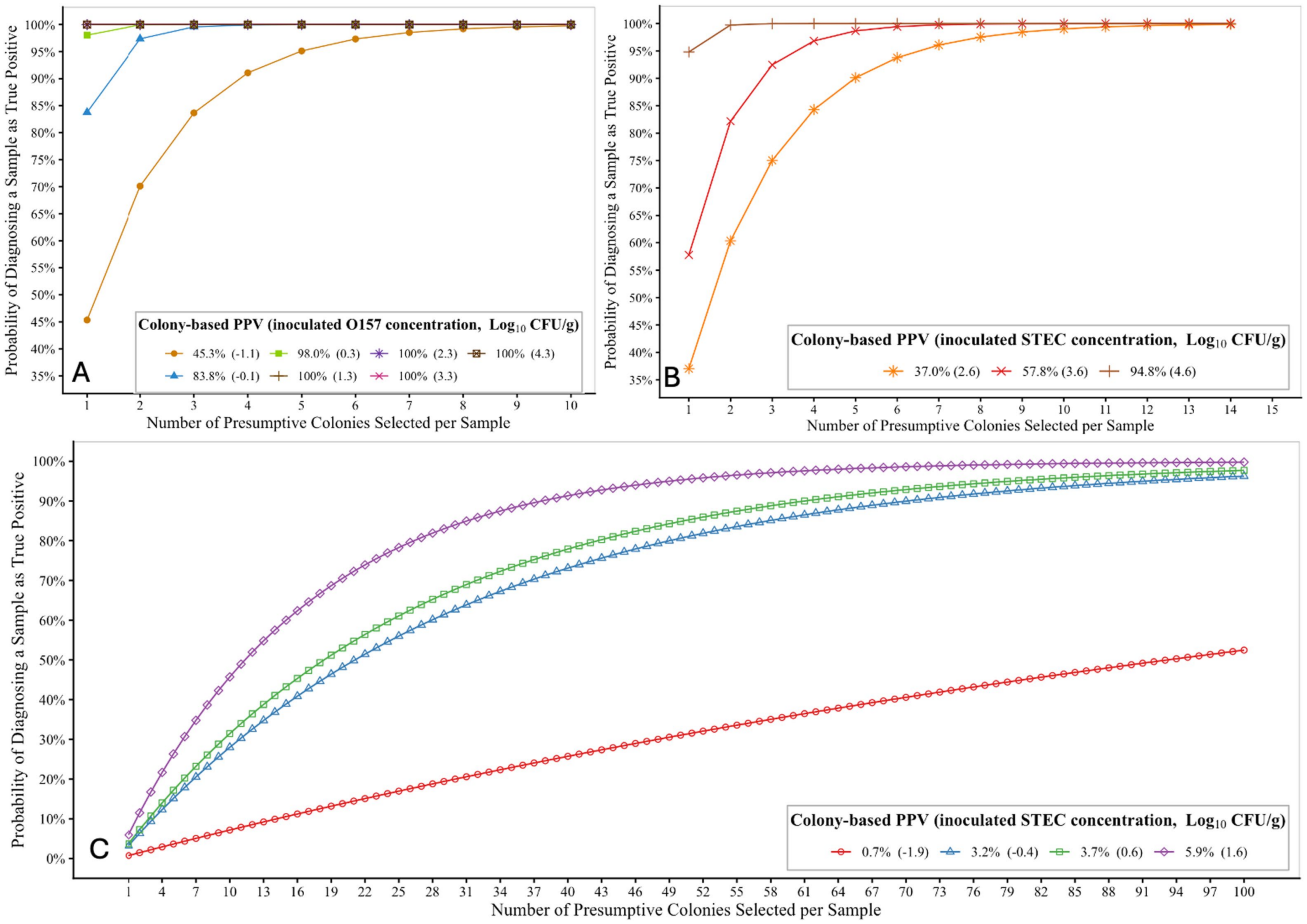
Probability of diagnosing a sample as true positive based on the number of selected presumptive colonies per sample, across varying concentrations in dairy manure samples for *E. coli* O157:H7 **(A)** and non-O157 STEC, [**(B)** for higher concentrations, **(C)** for lower concentrations]. O157 = *E. coli* O157:H7; STEC = non-O157 Shiga-toxin–producing of *E. coli*.

For *E. coli* O157:H7, at the lowest inoculation concentration (−1.1 log₁₀ CFU/g) which resulted in the c*PPV* = 45.3%, the probability of diagnosing a fecal sample as true positive reached 91% when only 4 presumptive colonies were selected (Figure 6A). In contrast, if only 4 selected colonies were chosen for non-O157 STEC, a comparable detection probability of >90% was only achieved at a substantially higher bacterial concentration (3.6 log₁₀ CFU/g) (Figure 6B). However, at concentrations ≤1.6 log₁₀ CFU/g for non-O157 STEC, many more presumptive colonies had to be selected for qPCR–confirmation to achieve a similar detection probability. For example, at concentration 1.6 log₁₀ CFU/g (which resulted in a c*PPV* ≤ 5.9%), a probability of 90% was achieved only after selecting up to 38 presumptive colonies (Figure 6C).

### 3.4 Estimation of fecal prevalence of *E. coli* O157:H7 and non-O157 STEC as a function of the number of presumptive colonies (x) selected for confirmation per sample from the longitudinal dairy fecal survey

Consistent with findings from the experimental trial, the number of presumptive colonies on selective agar plates varied for the two target pathogen groups for the samples obtained during the longitudinal dairy fecal survey. For *E. coli* O157:H7, this ranged from 0 presumptive colonies (observed in 17.9% of all samples; 25/140) to a maximum of 45 colonies (3.6% of all samples; 5/140). In contrast, for non-O157 STEC, all fecal samples had one or more presumptive colonies and the maximum of 45 colonies was observed for the majority of samples (87%; 122/140). The c*PPV*_sample_ for *E. coli* O157:H7 was 90 to ~100% in 32 of 53 observed positive samples (60.4%), indicating a low prevalence of false presumptive colonies given that over 90% were qPCR-confirmed in the majority of positive samples. In contrast, no fecal sample achieved a c*PPV*_sample_ ≥ 90% for non-O157 STEC; instead, only 4 samples fell into the range of 47–82% while the remaining samples were ≤40%. These low values for c*PPV*_sample_ for non-O157 STEC are indicative of the low specificity of the selective media; in other words, there is a low probability of finding a true positive despite the observation of one or more suspect colonies for these bacteria, especially at low concentrations of the target pathogens.

**Figure 7:**
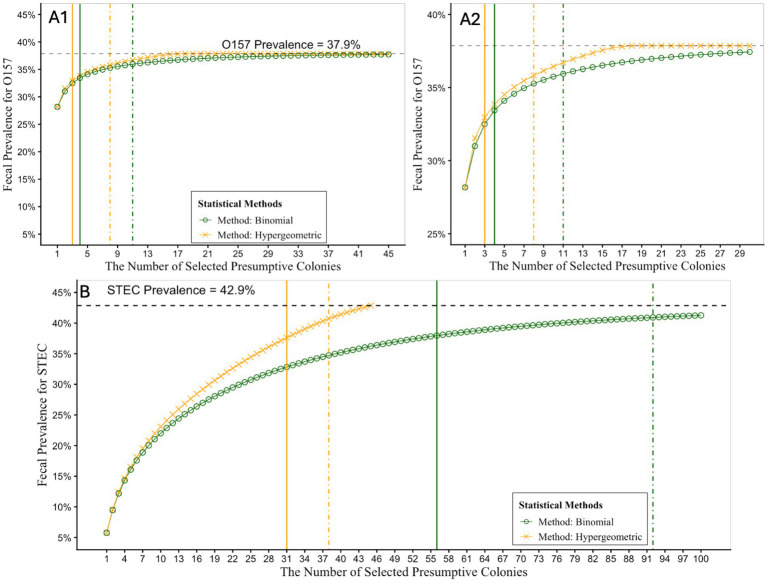
Fecal prevalence estimates for *E. coli* O157:H7 **(A1,A2)** and non-O157 STEC **(B)** in a dairy fecal longitudinal survey, calculated using hypergeometric and binomial statistical methods and plotted against the number of presumptive colonies selected for confirmation per sample. In **(A1)** and **(A2)** [a magnification of **(A1)**], the black dashed line represents the observed *E. coli* O157:H7 prevalence of 37.9% when using the 45-presumptive colony protocol. A maximum 2% allowable difference between the observed prevalence and model-predicted prevalence occurred when only 8 colonies (orange dot-dashed line, hypergeometric method) or 11 colonies (green solid line, binomial method) were selected. A maximum 5% allowable difference between the observed prevalence and model-predicted prevalence occurred when only 3 colonies (orange dot-dashed line, hypergeometric method) or 4 colonies (green solid line, binomial method) were selected. In **(B)**, the black dashed line represents the observed non-O157 STEC prevalence of 42.9%. A maximum 2% allowable difference between the observed prevalence and the model-predicted prevalence occurred when 38 colonies (orange dot-dashed line, hypergeometric method) or 92 colonies (green solid line, binomial method) were selected. A maximum 5% allowable difference between the observed prevalence and the model-predicted prevalence occurred when 31 colonies (orange dot-dashed line, hypergeometric method) or 56 colonies (green solid line, binomial method) were selected. O157 = *E. coli* O157:H7; STEC = non-O157 Shiga toxin-producing *E. coli*.

The overall observed prevalence of *E. coli* O157:H7 was 37.9% (53/140) and non-O157 STEC was 42.9% (60/140) when up to 45 presumptive colonies were selected without replacement for qPCR-confirmation per sample (i.e., a presumptive colony only sampled once if selected, referred to as the 45-presumptive colony protocol). Figure 7, which was calculated using Equations 6, 7, displays the full range of model-predicted prevalences when *x*_i_ is equal to 1 through 45 or more selected colonies for all 140 fecal samples from 14 California dairy farms. Our goal with these calculations was to model the effect of theoretically choosing different numbers of presumptive colonies per fecal sample on the model-predicted fecal prevalence for this set of 140 fecal samples as a case study. For example, if fewer presumptive colonies had been selected than the original target of up to 45 that generated the observed prevalences of 37.9 and 42.9% described above, there should be a tendency to underestimate the observed fecal prevalence due to inadvertently omitting from selection some of the original true positive colonies. For *E. coli* O157:H7 (Figures 7A1,A2), the model-predicted fecal prevalence when using the hypergeometric or binomial distribution ranged from 28.2 to 37.9% as the number of selected presumptive colonies per sample increased from 1 to 45. Setting a maximum allowable difference of 2% between the observed prevalence (37.9%) and the model-predicted fecal prevalence when choosing fewer colonies for confirmation (i.e., to save time or funding), this 2% maxima was achieved when selecting only 8 presumptive colonies based on the hypergeometric distribution and 11 colonies based on the binomial distribution. Alternatively, a 5% maximum allowable difference was achieved with selecting just 3 colonies based on the hypergeometric distribution and 4 colonies based on the binomial distribution. For non-O157 STEC (Figure 7B), the model-predicted fecal prevalence ranged from 5.8 to 42.9% as the number of selected presumptive colonies increased from 1 to 45, based on the hypergeometric distribution. In contrast, when using the binomial distribution (i.e., sampling colonies with replacement), the model-predicted fecal prevalence ranged from 5.8 to 36.2% over the same range of 1 to 45 selected colonies, and reached only 41.3% (and not the original observed prevalence of 42.9%) when extended to 100 selected colonies. A 2% maximum allowable difference between the observed prevalence (42.9%) and model-predicted prevalence was achieved when selecting 38 colonies (hypergeometric distribution) and 92 colonies (binomial distribution). A 5% maximum allowable difference was achieved with selecting 31 colonies (hypergeometric distribution) and 56 colonies (binomial distribution).

### 3.5 Conditions for convergence between hypergeometric and binomial predictions for the probability of diagnosing a sample as true positive

**Figure 8:**
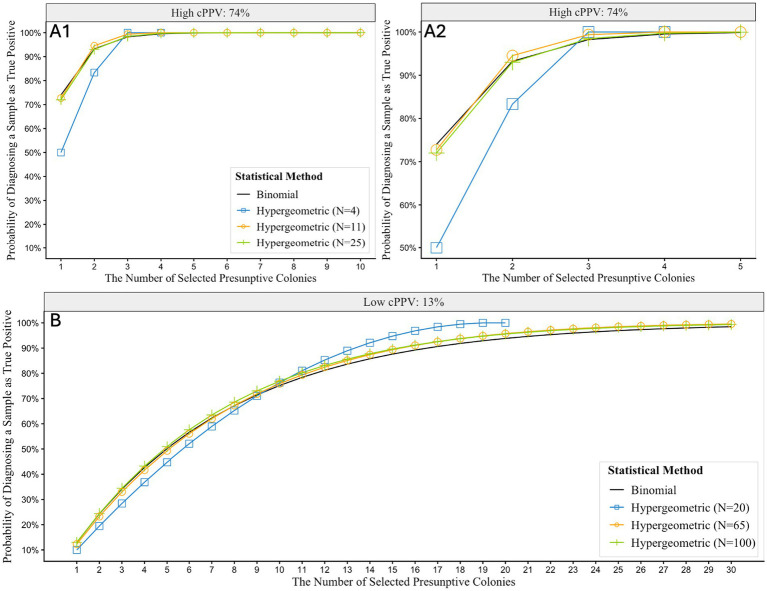
Probability of detecting at least one qPCR-confirmed positive colony (leading to the sample being classified as true positive) when selecting different numbers of presumptive colonies for confirmation, calculated using the binomial or hypergeometric distribution (with varying population sizes *N*) for two different colony-based positive predictive values (*cPPV*). Values for high *cPPV* = 74% [**(A1)**, with **(A2)** a magnification of **(A1)**] and low *cPPV* = 13% **(B)** were derived as the arithmetic mean of the *cPPV* for all positive samples for *E. coli* O157:H7 and non-O157 STEC from the dairy longitudinal survey, respectively. qPCR is quantitative-PCR. Non-O157 STEC = non-O157 Shiga toxin-producing *E. coli*.

From Figure 7, fecal prevalence estimates derived from the binomial (Pr_BN_) and hypergeometric (Pr_HG_) models were relatively similar for *E. coli* O157:H7 across the range of 1 to 45 selected presumptive colonies. In contrast, substantial differences were observed for the fecal prevalence estimates between the two models for non-O157 STEC across the range of selected presumptive colonies, indicating considerable divergence for the estimates generated by the binomial and hypergeometric models for the probability to diagnose a sample as true positive (Pr_HG_ and Pr_BN_). The arithmetic mean c*PPV_sample_* for all observed positive samples from the dairy longitudinal survey was 74% for *E. coli* O157:H7 (relatively high c*PPV*) and 13% for non-O157 STEC (relatively low c*PPV*). These high and low c*PPV* values were then used to explore convergence conditions between the binomial (Equation 4) and hypergeometric (Equation 5) models under varying presumptive colony population sizes (*N*) per agar plate and selected colonies for confirmation (*n*). Recall that *N* is needed to make hypergeometric probability calculations, but this parameter is often unknown or unknowable, hence, the concern over assuming its value. For the high c*PPV* scenario (74%; Figures 8A1,A2), *N* had to be greater than or equal to 11 presumptive colonies per agar plate to achieve this maximum allowable difference of 2%. Assuming there are at least 11 presumptive *E. coli* O157:H7 colonies on a positive agar plate following enrichment is not an unreasonable expectation and easily verified through confirmation given the low count. Reducing *N* to 4 from 11 did not result in a noticeable discrepancy between the models given that both Pr_HG_ and Pr_BN_ approached ~100% at *N* = 4 and remained high even at *N* = 2 (Pr_HG_ = 95%, Pr_BN_ = 93%). Therefore, a laboratory protocol recommending no more than *n* = 4 selected presumptive colonies per fecal sample for *E. coli* O157:H7 using the diagnostic methods and selective agar as outlined above would have been resource efficient for these 140 dairy fecal samples while simultaneously minimizing false negatives.

For the low c*PPV* scenario (13%; Figure 8B), a 2% convergence criteria required a substantially larger *N* of ≥65. Reducing *N*, for example, to 20 generated a substantial divergence between Pr_HG_ and Pr_BN_ when *n* ranged from 15 to 20 (*n* = number of selected colonies out of *N* total presumptive colonies, with *n* ≤ *N*). Selecting *n* = 12 presumptive colonies when *N* ≥ 20 would be sufficient for matrices such as dairy cattle feces if probabilities ≥80% are acceptable for detecting a true positive. Given the low specificity of non-O157 STEC agar, *N* is typically much larger than 20 presumptive colonies per agar plate. In order to achieve a detection probability ≥90%, at least *n* = 17 presumptive colonies must be selected. In contrast to the high c*PPV* scenario above for *E. coli* O157:H7, selecting only 4 colonies resulted in substantially lower detection probabilities (Pr_HG_ ≈ 42%, Pr_BN_ ≈ 43%) that are likely insufficient for most research purposes since there is a > 50% chance of classifying a fecal sample as false negative.

## 4 Discussion

Our modeling approach focused on using the proportion of presumptive colonies that confirm positive by qPCR (c*PPV*) in combination with the total number of selected colonies per agar plate to demonstrate how these variables influence (1) the probability to detect a true positive among suspect samples and (2) the observed prevalence of true positive samples. These probability models (Pr_BN_ and Pr_HG_) demonstrated that the number of presumptive colonies selected for confirmation per sample was a key factor influencing an assay’s ability to detect a true positive sample, especially for CHROMagar STEC which exhibited low specificity given the low values observed for c*PPV* for both the experimental trial and the dairy longitudinal study (i.e., 13%). This observation of low specificity for CHROMagar STEC has been widely reported (Gouali et al., 2013; McCallum et al., 2013; Wylie et al., 2013; Lubeskie et al., 2016; Zelyas et al., 2016). In addition, as might be expected for the *PPV* of any assay targeting specific microorganisms in a fixed mass or volume of sample, the c*PPV* values for the selective media targeting either *E. coli* O157:H7 and non-O157 STEC were highly conditional on the underlying concentration of the target bacteria, as shown in Figure 5.

Regarding non-O157 STEC selective media, c*PPV* values were quite low (<10%) for the concentrations below 2 log_10_ CFU/g feces, indicating that over 90% of the presumptive colonies were false positives. Given these low levels for c*PPV* for non-O157 STEC selective media, it is readily predicted by Pr_BN_ and Pr_HG_ (i.e., probability to detect true positive) that a disproportionally high number of presumptive colonies will need to be selected for confirmation in order to find a true positive colony among the many false presumptive colonies. This lack of specificity for non-O157 STEC selective media results in greater variability for the number of presumptive colonies selected by each of three laboratory technicians once bacterial concentrations exceeded 2 log_10_ CFU/g feces; apparently, each technician varies in their interpretation of what constitutes non-O157 STEC presumptive characteristics at the colony level (Figure 4). In contrast, the c*PPV* values for the *E. coli* O157:H7 selective media were considerably higher at almost any spiked concentration compared to c*PPV* values for non-O157 STEC selective media, with c*PPV* values approaching 100% once the bacterial concentration was ≥0.3 log₁₀ CFU/g (Figure 5). These high c*PPV* values are indicative of the higher specificity of this agar, necessitating the selection of far fewer presumptive colonies in the effort to find a true positive.

The primary goal of our study was to determine an optimal number of presumptive colonies to select per sample from fecal matrices that generated both a high probability to detect true positive samples and an accurate estimate of STEC prevalence in feces, all the while minimizing costs associated with selecting excessive numbers of presumptive colonies. Our study indicated that the high c*PPV* (~100%) values at all but the lowest bacterial concentration (−1.1 log₁₀ CFU/g) for the combination of CT-SMAC and Rainbow Agar O157 generated a > 90% probability of true positive detection when as few as 4 presumptive colonies were selected (Figure 6). Similarly, choosing as few as 3-to-4 presumptive colonies achieved a reasonably accurate estimate of the fecal prevalence of *E. coli* O157:H7 for the dairy longitudinal study (Figure 7).

In contrast, regarding CHROMagar STEC, a considerably larger number of presumptive colonies need to be selected for confirmation in order to detect fecal samples positive for non-O157 STEC. In order to achieve a ≥ 90% detection probability, a technician would need to select a minimum of 38 colonies per sample when the maximum concentration of bacterial was ≤1.6 log₁₀ CFU/g. If we knew *a priori* that the expected non-O157 STEC concentrations would be ≥2.6 log₁₀ CFU/g, as few as 5 presumptive colonies would generate a similar detection probability of ≥90%, but we generally do not know beforehand what the frequency distribution of CFU/g of the target pathogen is in a new set of fecal samples. Despite these limitations, many researchers continue to favor CHROMagar STEC to detect non-O157 serogroups of public health importance (e.g., O26, O45, O103, O111, O121, and O145) due to several well-documented advantages, such as ease of preparation, extended shelf life; strong performance in detecting non-O157 STEC from stool cultures, and the ability to identify a broad range of non-O157 STEC serotypes while variably suppressing background fecal flora (Gouali et al., 2013; McCallum et al., 2013; Wylie et al., 2013; Kase et al., 2015; Zelyas et al., 2016). Alternatively, as recommended by Zelyas et al. (2016) and Jenkins et al. (2020), CHROMagar STEC could be used to recover isolates from specimens that have already tested positive for non-O157 STEC or combined with other selective media (Zelyas et al., 2016; Feng et al., 2018; Jenkins et al., 2020).

The model predictions shown in Figures 6–8 substantially rely on the use of the hypergeometric and binomial distributions to model the probability the detecting a true positive fecal sample as a function of confirming one or more presumptive colonies, with these calculations then used for an estimate of the STEC prevalence for a set of fecal samples. Valid use of the hypergeometric distribution for this application requires that a laboratory technician sample presumptive colonies without replacement; in other words, presumptive colonies on an agar plate are sampled only once for confirmation, as is common practice. As a consequence, the probability of detecting a true positive colony (*k*/*n*) among all remaining presumptive colonies on an agar plate increases with each successive pick of a false positive presumptive colony (*k*-*n*) that is then removed from further selection. In contrast, valid use of the binomial distribution for this application expects a laboratory technician to sample presumptive colonies with replacement. For example, assume a technician intends to pick 5 presumptive colonies for confirmation on an agar plate; then for each round of selecting a presumptive colony, all presumptive colonies are equally likely to be sampled for each round, which equates to *p* = *k*/*n* (probability of selecting a true positive colony) remaining the same for each round of colony selection. This requirement for sampling presumptive colonies with replacement when using the binomial distribution is not normal practice in diagnostic microbiology and therefore a limitation of this probability distribution. Furthermore, as shown in Figures 6, 7, when c*PPV* is low and/or the concentration of the target microorganism is low, the binomial distribution substantially under-estimates the probability to detect true positive samples and the associated prevalence. Despite these shortcomings, the binomial distribution for this and other similar applications can nicely approximate the hypergeometric probability predictions under certain circumstances, all the while avoiding the necessity required by the hypergeometric distribution to estimate *K* (total number of true positive colonies on an entire agar plate) and *N* (total number of presumptive colonies on an entire agar plate). For this study, the probability estimates derived from the binomial model (Pr_BN_) approximate those from the hypergeometric model (Pr_HG_) when the sampled fraction (*n*/*N*) is small (<10%) (Devore, 2016). This convergence between the two distributions is particularly relevant when the number of selected presumptive colonies (*n*) constitutes only a small subset of the total available presumptive colonies (*N*) on an agar plate; a situation that frequently occurs under the following conditions: (1) the target bacterium is present at high or too-numerous-to-count concentrations in the sample (high *N*), and (2) the selective agar has relatively low specificity (low c*PPV*), leading to a large number (high *N*) of suspect presumptive colonies (a common feature of media designed for non-O157 STEC detection). In addition, the analyses from this work demonstrate that when c*PPV* is relatively high, even a small number of selected colonies (*n*) are sufficient for the binomial and hypergeometric estimates to converge closely regardless of our assumptions regarding *N* (as shown in Figures 7A1,A2, 8A1,A2 for the high c*PPV* scenario).

The specific recommendations presented in this study are limited to the protocol and matrices evaluated herein. Caution should therefore be exercised when applying these findings to other sample matrices. Optimal numbers of presumptive colonies chosen for confirmation are protocol- and matrix-specific and should be re-modeled for different settings. Nevertheless, the modeling approach is generalizable: as long as the c*PPV*, ratio of *K* and *N* are the same, the predicted probability are the same even under different matrices and protocols as they are derived from the same mathematical formulas. The 90% probability of diagnosing a sample as true positive, and maximum allowable differences of 2 and 5% different from observed prevalence, were each selected arbitrarily but reasonably acceptable by various researchers. Investigators may choose different thresholds according to their specific objectives, acceptable levels of error, and available resources, noting that more stringent criteria require the selection of additional presumptive colonies. Overall, this manuscript does not constitute a full method validation. Rather, it demonstrated the substantial impact of the number of presumptive colonies selected per sample on the probability of correctly identifying a sample as truly positive and, ultimately, on STEC prevalence estimates.

## 5 Conclusion

Using presumptive colonies as the unit of analysis, our study demonstrated that pathogen concentration significantly influenced c*PPV*. Furthermore, the value of c*PPV*, when combined with the number of selected presumptive colonies, directly influenced the probability to detect a true positive fecal sample and the associated prevalence of STEC in a set of samples. For *E. coli* O157:H7, a 91.1% probability of true-positive detection was achieved with only 4 presumptive colonies due to the high specificity of the selective agars resulting in a high c*PPV* value, even at the low concentrations (−1.1 log₁₀ CFU/g). Selecting 3–4 presumptive colonies produced a model-predicted prevalence of ~33% (difference <5% from the observed 37.9%). The difference fell to <2% with 8 (HG) or 11 (BN) colonies. In contrast, a similar protocol of just 4 presumptive colonies for non-O157 STEC would likely perform poorly at concentrations less than 2.6 log₁₀ CFU/g, in part due to the low specificity of the selective agar leading to low c*PPV* values. For non-O157 STEC concentrations ≤1.6 Log₁₀ CFU/g, achieving >90% detection probability requires a minimum of 38 selected presumptive colonies per sample. Model predictions for non-O157 STEC reached ~38% with 31 colonies for the HG model and 56 colonies for the BN model, differing by 5% from the observed prevalence (42.9%). This difference decreased to <2% when 38 colonies were selected for the HG model; in contrast, this level accuracy requires at least 92 colonies be selected for the BN model. These numbers are impractically high for many routine laboratory endeavors, but would be unavoidable for samples expected to have low target pathogen concentrations. Improving the specificity of the selective agar and/or improving protocols for selective enrichment of non-O157 STEC would together improve assay performance for this group of bacterial pathogens. To improve clarity, reproducibility, and comparability across studies, we recommend research reports to include the total number of presumptive colonies selected per sample when assessing the prevalence of *E. coli* O157:H7 and especially non-O157 STEC.

## Data Availability

The raw data supporting the conclusions of this article will be made available by the authors, without undue reservation.
